# Development and Validation of Four Different Methods to Improve MRI-CEST Tumor pH Mapping in Presence of Fat

**DOI:** 10.3390/jimaging10070166

**Published:** 2024-07-12

**Authors:** Francesco Gammaraccio, Daisy Villano, Pietro Irrera, Annasofia A. Anemone, Antonella Carella, Alessia Corrado, Dario Livio Longo

**Affiliations:** 1 Department of Molecular Biotechnology and Health Sciences, University of Turin, 10126 Torino, Italy; 2 Institute of Biostructures and Bioimaging (IBB), National Research Council of Italy (CNR), 10126 Torino, Italy

**Keywords:** MRI, chemical exchange saturation transfer (CEST), fat, tumor, pH, acidosis, iopamidol, contrast agent, pH responsive

## Abstract

CEST-MRI is an emerging imaging technique suitable for various in vivo applications, including the quantification of tumor acidosis. Traditionally, CEST contrast is calculated by asymmetry analysis, but the presence of fat signals leads to wrong contrast quantification and hence to inaccurate pH measurements. In this study, we investigated four post-processing approaches to overcome fat signal influences and enable correct CEST contrast calculations and tumor pH measurements using iopamidol. The proposed methods involve replacing the Z-spectrum region affected by fat peaks by (i) using a linear interpolation of the fat frequencies, (ii) applying water pool Lorentzian fitting, (iii) considering only the positive part of the Z-spectrum, or (iv) calculating a correction factor for the ratiometric value. In vitro and in vivo studies demonstrated the possibility of using these approaches to calculate CEST contrast and then to measure tumor pH, even in the presence of moderate to high fat fraction values. However, only the method based on the water pool Lorentzian fitting produced highly accurate results in terms of pH measurement in tumor-bearing mice with low and high fat contents.

## 1. Introduction

Chemical exchange saturation transfer (CEST) magnetic resonance imaging (MRI) is a novel approach in the field of molecular imaging used to investigate small molecules [1,2,3]. MRI-CEST imaging can exploit endogenous molecules naturally present in the human body or exogenous molecules to enable the precise mapping and quantification of tumor pH [4,5,6,7,8,9,10,11,12,13,14,15,16,17]. Among these approaches, iopamidol-based tumor pH imaging is an established one that offers high accuracy, sensitivity and spatial resolution in quantifying extracellular tumor pH across a diverse range of tumor types [18,19]. In addition, tumor pH imaging based on iopamidol has been exploited in several investigational studies to assess the therapeutic efficacy of a large variety of drugs targeting different aspects of tumor metabolism, acidosis, and immunotherapies [18,19,20,21,22,23,24,25,26,27,28,29,30,31]. Of note, the clinical translation of pH imaging based on iopamidol or on similar pH-responsive molecules demonstrates the urgent need for an accurate pH imaging approach at the bedside [32,33,34,35,36,37,38,39,40].

Iopamidol is an iodinated contrast agent known for its two distinct amide groups with varying resonance frequencies, specifically at 4.2 ppm and 5.5 ppm [41]. The CEST effect at a specific offset is calculated using asymmetry analysis by comparing the water signal upon irradiation at specific offset(s) and at the opposite (negative) frequency offset (for iopamidol, ∆ω = 4.2 ppm and 5.5 ppm). One of the challenges posed by asymmetry analysis is the presence of artifacts caused by lipid signals [42]. In fact, the presence of a fat signal might result in the inaccurate normalization of the Z-spectrum, consequently creating an intrinsic dependence of CEST signals on the fat content within each voxel [43].

This dependence on fat content becomes particularly relevant in CEST investigations targeting tissues with substantial fat contents, such as those commonly observed in breast cancer cases, mammary glands and other adipose tissues [44,45,46,47,48,49,50,51,52,53,54,55,56,57,58,59,60,61,62,63]. The fat signal within the fibroglandular breast tissue can affect CEST quantification [64], and careful analysis is therefore required to obtain accurate calculations, including pH measurements [65]. Several algorithms and denoising methods have been investigated to improve CEST contrast quantification due to the inherently low signal-to-noise ratio and susceptibility to image noise of CEST images, although not addressing the presence of strong lipid signals [66,67,68,69,70,71,72,73,74,75,76,77].

Several authors have proposed different methods to remove lipid artifacts from conventional MRI images by adopting several fat suppression strategies for various MRI examinations [78,79,80,81,82,83,84,85,86,87,88]. Within the MRI-CEST field, alternative acquisition strategies and fat suppression schemes have been exploited [89,90,91,92]. Sun et al. investigated echo-planar imaging, which revealed the presence of a lipid artifact in Amide Proton Transfer (APT) asymmetry images, and they were able to address this issue effectively by employing a combination of a chemical-shift-selective refocusing pulse and crusher gradients, resulting in the production of high-quality images [93]. In a study conducted by Lu et al., the authors successfully achieved lipid suppression using a Gaussian-shaped chemical-shift-selective pulse with a 90° flip angle and a duration of 6 milliseconds. The frequency offset of this lipid suppression pulse was set at −3.5 ppm relative to the water resonance frequency. This specific offset effectively suppressed most lipid signals while leaving the water signal unaffected [94]. In the study by Zhang et al., the authors proposed an approach based on the interplay among the echo time (TE), fat fraction (*FF*) and Z-spectrum to remove the fat contribution to CEST contrast quantification [95,96]. Moreover, a multi-echo Dixon technique with a self-adapting multi-peak model has been validated in the fibroglandular region of the breast to remove lipid artifacts in CEST imaging [97]. In a similar way, multi-echo-based fat–water separation with an adaptive fat model that utilizes the full complex data and a phase demodulation approach was exploited for robust fat artifact correction [91]. Recently, a post-processing technique has been introduced for the fat correction of APT and guanidyl CEST contrast to differentiate between normal-appearing fibroglandular tissue and breast tumors [98]. Within this approach, a multi-Lorentzian fit analysis was developed to increase the diagnostic accuracy of the calculated relaxation-compensated and fat-corrected CEST contrast.

The objective of this study is to compare four different methods to quantify the CEST contrast by removing artifacts arising from fat signals without the need to apply complex fat saturation or fat–water separation techniques but applying only post-processing procedures. The four methods were validated in vitro and in vivo on two different tumor models with a low or high amount of fat by comparing the obtained tumor extracellular pH maps.

## 2. Materials and Methods

### 2.1. Theory

The CEST effect is quantified by examining the water signal’s intensity when subjected to irradiation at two distinct frequency offsets from the resonance point of bulk water, which is set at 0 ppm. This method is crucial to account for any symmetric influences on the water signal resulting from the application of the irradiation pulse.

Traditionally, the saturation transfer (*ST*) efficiency is quantified at a specific offset using asymmetry analysis (also dubbed Magnetization Transfer Ratio asymmetry, or MTRasym):

(1) $$ST=\frac{S_{-∆\omega }-S_{\;∆\omega }}{S_{-∆\omega }}$$

where $S_{\pm ∆\omega }\;$ is the water signal intensity in the presence of a saturation pulse at an offset of ±∆ω (i.e., Δω = 4.2 ppm and 5.5 ppm for iopamidol).

**Figure 1:**
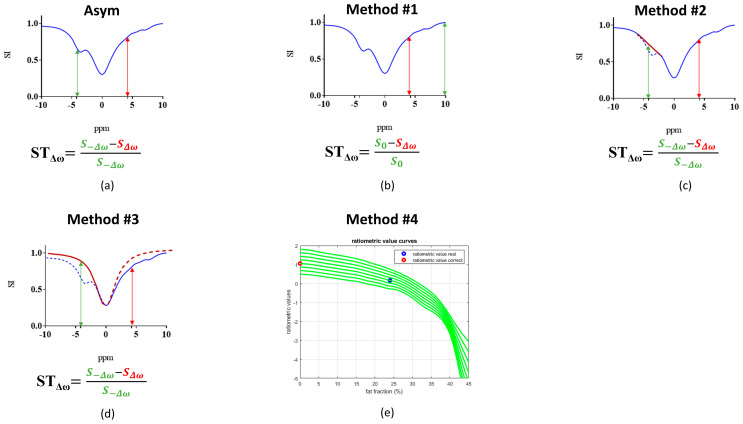
Graphical representation of a representative simulated Z-spectrum in blue (signal intensity: SI as a function of the irradiation offset in ppm) and of CEST contrast quantification by applying asymmetry analysis or the other three investigated methods. (**a**) Conventionally, saturation transfer (*ST*) efficiency is quantified at a specific offset using asymmetry analysis (double arrow in red at +4.2 ppm and double arrow in green at −4.2 ppm). (**b**) Method #1: The CEST contrast is calculated considering only the frequency offsets in the positive part of the Z-spectrum. (**c**) Method #2: Z-spectra offsets centered around the fat signal are replaced with a linear interpolation (red line). (**d**) Method #3: The negative part of the Z-spectrum is replaced by the water pool contribution upon Lorentzian fitting of the Z-spectrum (red curve). (**e**) Method #4: The calculated set of interpolated curves (green lines) of fat-corrected ratiometric values. The blue circle represents the measured ratiometric value with a fat fraction of 25%, and the red circle represents the corrected ratiometric value in the absence of fat (fat fraction = 0%).

The CEST contrast is significantly influenced by the overlapping fat signal when fatty tissue is present. The first three approaches were developed by exploiting the Z-spectrum shape properties (Figure 1), whereas the fourth method relies on the application of a correction factor to adjust the ratiometric values according to the percentage of fat:

- Method #1 calculates the contrast considering only the positive part of the Z-spectrum using the following equation:
  (2) $$ST=\frac{S_{0}-S_{\;∆\omega }}{S_{0}}$$
  where $S_{∆\omega }$ is the water signal intensity in the presence of a saturation pulse at an offset ∆ω, and $S_{0}$ is the water signal intensity without any saturation.
- Method #2 consists of removing the fat frequencies (in the range of −2 to −5.9 ppm) and replacing the missing range with a linear interpolation. The CEST contrast is then calculated by asymmetry analysis using equation #1.
- Method #3 consists of replacing the negative part of the Z-spectrum with the water pool contribution upon Lorentzian fitting of the spectrum, and the contrast is then calculated by asymmetry analysis using the same equation #1.
- Method #4 corrects the calculated ratiometric values according to the measured fat fraction levels by interpolating the ratiometric values with cubic splines to correct for the proper pH values in the absence of fat (more details in the Supplementary Materials).

### 2.2. In Vitro MRI Studies

**Figure 2:**
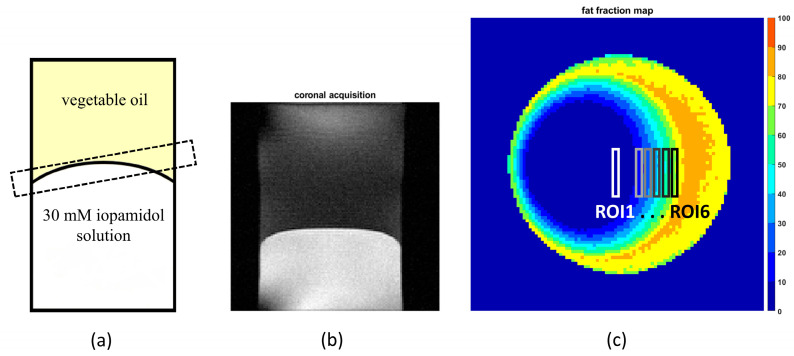
For in vitro validation, a 50 ml Falcon tube phantom was prepared consisting of a solution containing 30 mM iopamidol (titrated at pH = 6.9) layered with sunflower oil. (**a**) The phantom diagram illustrates the oblique placement of the slice, where the interface area shows the gradual mixing of fat and water within the slice. (**b**) MRI T2w-coronal image of the phantom showing the iopamidol-containing water (bottom) and a vegetable oil (top) solutions. (**c**) The fat fraction map of the section shows a linear gradient across the interface. The regions of interest (ROIs) are arranged from left to right, each corresponding to a specific range of the fat fraction in increments of 10%. ROI 1 includes pixels with a fat fraction between 0 and 10%, up to ROI 6, which includes pixels with a fat fraction between 50 and 60%.

Two phantoms were prepared for in vitro validation. The phantoms consisted of a 50 mL Falcon conical tube (diameter of 30 mm and length of 115 mm) filled with 1X phosphate-buffered saline (PBS) solution containing 30 mM iopamidol, one with the pH adjusted to 6.4 and the other with the pH adjusted to 6.9. The solution was then layered with sunflower oil. To create a gradient in the fat fraction across the imaging plane, the imaging plane was angled at the interface of the two liquid phases of the sample (Figure 2) [43].

The fat fraction (*FF*) was estimated as follows:

(3) $$FF=\frac{F}{F+W}$$

where *F* and *W* are the fat and water peak amplitudes [99].

Z-spectra were acquired on a Bruker Avance 300 operating at 7 T (Bruker BioSpin MRI, Ettlingen, Germany) equipped with a MICWB40 RES 1H 040/030 QTR imaging probe. The scan protocol consisted of a CEST sequence (TR, 3.247 s; TE, 3.768 ms; NEX, 1; rare factor, 64; field of view [FOV], 3.5 × 3.5 cm; slice thickness, 3 mm; matrix, 96 × 96; the RF saturation offset was varied between ±10 ppm at intervals of 0.1 ppm) and a Dixon sequence (TR, 4s; TE, 9.75 ms; NEX, 1; rare factor, 8; field of view [FOV], 3.5 × 3.5 cm; slice thickness, 3 mm; matrix, 128 × 128).

### 2.3. In Vivo MRI Studies

Two breast cancer murine models were exploited because they were characterized by low (4T1, triple-negative breast cancer cells) and high (MMTV-PyMT, transgenic breast murine model) amounts of fat. Female BALB/c mice (Charles River Laboratories Italia S.r.l., Calco, Italy) and female MMTV-PyMT mice (The Jackson Laboratory, Bar Harbor, ME, USA) were maintained in the animal facility of the Dept. of Molecular Biotechnology and Health Sciences, University of Turin, under specific pathogen-free conditions. Animal manipulation and experimental procedures were carried out in accordance with the European Community guidelines (directive 2010/63) and under the approval of the Italian Ministry of Health (authorization #741/2022 and #958/2018). A 6-week-old female BALB/c mouse was subcutaneously inoculated with 4T1 (1 × 10^6^ cells) resuspended in 50 μL of phosphate saline buffer (PBS, Sigma Aldrich, Milano, Italy) in both 4th mammary glands’ fat pads, whereas a female FVB/N-Tg(MMTV-PyVT) transgenic mouse (PyMT) spontaneously developed tumors that were visible after four weeks. Before MRI acquisition, mice were anesthetized by an intramuscular injection of a mixture of 5 mg/kg of xylazine (Rompun, Bayer, Italy) and 20 mg/kg of tiletamine/zolazepam (Zoletil 100, Virbac, Italy), and a 27-gauge needle was introduced into the tail vain for contrast agent injection. During the acquisition, the breath rate was monitored by an air pillow placed below the animal (SA Instruments, Stony Brook, NY, USA). CEST pH mapping was performed upon the intravenous injection of 4 g I/kg b.w. of iopamidol (Isovue370, kindly provided by Bracco Imaging SpA, Colleretto Giacosa, Italy).

MR images were acquired with a Bruker 7T Avance 300 MRI scanner (Bruker Biospin, Ettlingen, Germany) using a 30 mm insert coil. After a scout acquisition, anatomical T2w images were acquired with a fast spin-echo RARE sequence (repetition time (TR) = 4000 ms, echo time (TE) = 5.79 ms, number of slices = 8, slice thickness = 1.5 mm, FOV = 30 mm; matrix = 256 × 256, two averages, acquisition time = 2.56 ms), and the same geometry was used for CEST acquisition. The Z-spectra of CEST-MRI were acquired using a single-shot RARE sequence with centric encoding (typical setting TR/TE = 12,000 s/3.76 ms) preceded by a 3 µT cw block presaturation pulse and by a fat-suppression module [100]. A series of 46 MR frequencies were saturated to acquire a CEST spectrum in the frequency offset range ±10 ppm. We used an acquisition matrix of 96 × 96 reconstructed to 128 × 128 for a field of view of 3 × 3 cm^2^ (in-plane spatial resolution = 234 µm) with eight slices to cover the whole tumor with a slice thickness = 1.5 mm [101]. MRI-CEST image acquisition was repeated before and after the i.v. injection of iodinated contrast media (dose = 4 g iodine/kg body weight). The total scan time for CEST pH imaging was approximately 20 min. Fat quantification was performed with a Dixon method implemented in Paravision 360.1 by Bruker with the following parameters: TR = 4 s; TE = 39.4 ms; number of slices = 8; slice thickness = 1.5 mm; FOV = 30 mm; matrix = 128 × 128; three averages. The total acquisition time was approximately 6 min.

### 2.4. Data Analysis

The data were processed using an in-house script written in MATLAB R2023b (The MathWorks, Inc., Natick, MA, USA): https://github.com/cim-unito/Fat-Correction (accessed on 24 June 2024). Lorentzian CEST curve fitting was implemented with the open-source Matlab-based code (https://github.com/cest-sources/CEST_EVAL, last accessed on 15 October 2023) [102,103,104,105].

Prior to commencing data analysis, a background removal process was performed. A smoothed cubic spline algorithm was employed to perform interpolation on the Z-spectrum. B0-field shifting was determined by measuring the distance from the minimum point of the interpolated Z-spectrum by cubic smoothing splines to 0 ppm. The saturation transfer (*ST*) efficiency was quantified at a specific offset using Equation (1) or (2). Ratiometric values (*R_ST_*) were determined by dividing the contrast values obtained at two distinct offsets utilizing the following equation:

(4) $$R_{ST}=\frac{{ST}_{4.2\;ppm}}{{ST}_{5.5\;ppm}}$$

Voxel-wise pH maps were generated by extrapolating pH values from the ratiometric curves.

## 3. Results

**Figure 3:**
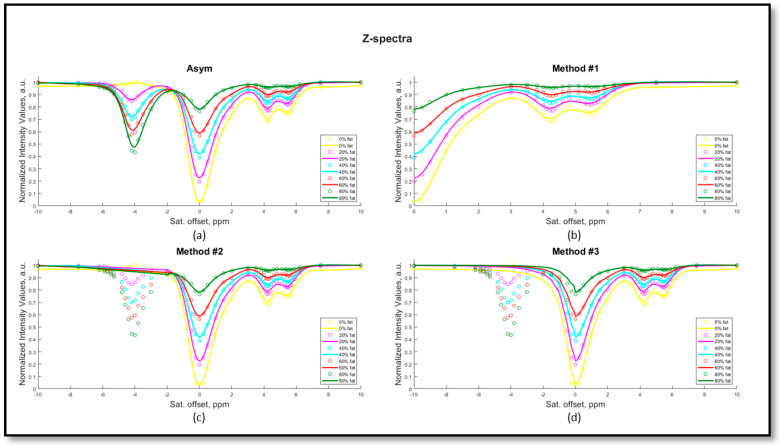
In vitro Z-spectra of iopamidol 30 mM titrated at pH = 6.9 at several percentages of fat values in the range 0–80% and graphical representations of the proposed methods for fat correction. The asymmetry analysis utilizes the original Z-spectrum (**a**), method #1 considers only the positive part of the Z-spectrum (**b**), method #2 replaces the negative part of the Z-spectrum with a linear interpolation (**c**) and method #3 consists of replacing the negative part of the Z-spectrum with the water pool contribution upon Lorentzian fitting of the spectrum (**d**).

**Figure 4:**
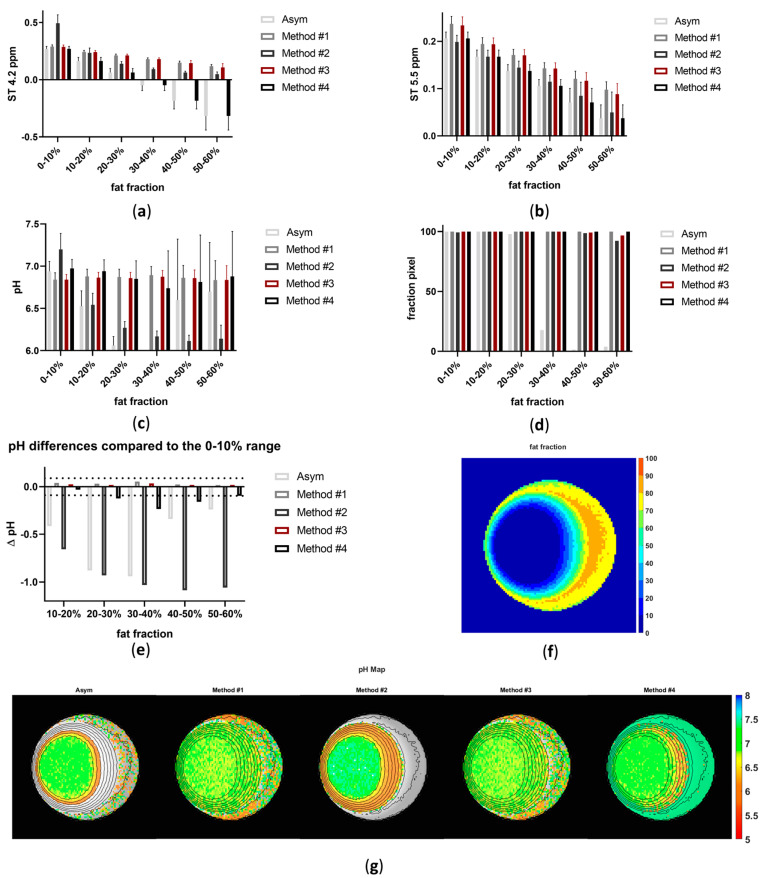
In vitro *ST*_4.2ppm_ (**a**), *ST*_5.5ppm_ (**b**), mean pH values at different fat fractions (**c**), percentage of detected pixels (**d**), pH differences (ΔpH) compared to the 0–10% range across different fat fraction ranges (dashed lines correspond to ±0.1 pH unit difference) (**e**), fat fraction map (**f**) and pH map (**g**), obtained with the different approaches with the phantom at pH 6.9.

The acquired Z-spectra for iopamidol in the presence of several fat fraction values and the corresponding modified Z-spectra using the various proposed methods to correct for the fat signal are depicted in Figure 3a for the iopamidol solution at pH 6.9 and in Figure S5 for the iopamidol phantom titrated at pH 6.4. The amplitude of both the water and iopamidol resonance peaks is strongly affected by the presence of fat. Moreover, beyond a certain threshold of fat content, the detectability of the CEST contrast arising from the two iopamidol amide peaks (resonating at 4.2 and 5.5 ppm) is markedly reduced, decreasing to negative values when *FF* values are greater than 30% (Figure 4a,b). Consequently, increased fat fraction values strongly affect the corresponding pH calculation (Figure 4c,e) and the fraction of pixels where reliable pH values can be calculated (Figure 4d,f), even in the presence of fat suppression schemes. In this scenario, asymmetry analysis enables pH measurement only up to an *FF* less than 10%, since for *FF* values already exceeding 10%, there is a tendency for the pH measurement to be underestimated (Figure 4c) or not calculated at all (Figure 4f).

The linear method (#2) overestimates the correct pH values when fat fractions are above 10% and underestimates pH values when *FF* values exceed 20%. Both the positive method (#1) and the Lorentzian method (#3) measure comparable CEST contrast for fat fraction values up to 40–50%, proving to be the most robust to increasing fat signals (Figure 4e). Moreover, both positive and Lorentzian methods (#1 and #3) provide robust and accurate pH measurements up to *FF* values of 40–60% (Figure 4c). The proposed interpolation method (#4) showed reduced CEST contrast quantification for *FF* values above 40%, with robust pH measurements for *FF* values in the *FF* range of 0 to 30%. Of note, both the positive and Lorentzian methods (#1 and #3) were able to increase the number of pixels where pH was measurable, with more accurate pH values (Figure 4d,g).

**Figure 5:**
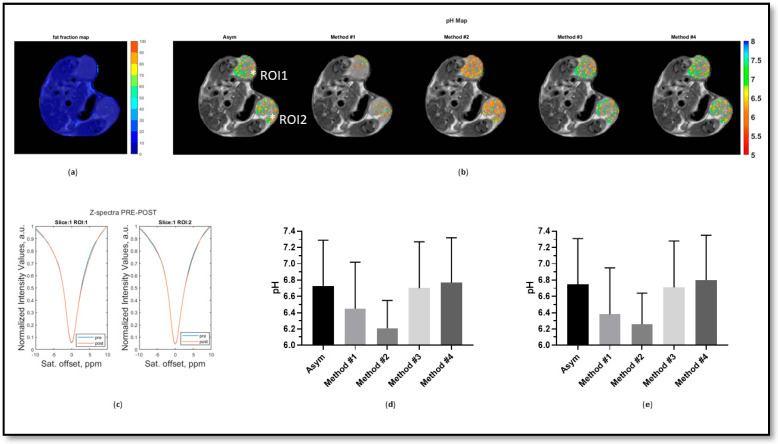
The fat fraction map (**a**), calculated tumor pH maps (**b**), Z-spectra before (blue) and after (red) iopamidol injection for the two ROIs (**c**), and tumor pH mean ± standard deviation values for ROI 1 (**d**) and ROI 2 (**e**), calculated for all investigated methods in a tumor-bearing mouse with a low amount of fat.

The four methods were also validated in vivo on two different tumor models characterized by low and high amounts of fat. The 4T1 tumors showed low to moderate amounts of fat (ca. 3% and 4% for RO1 and RO2, respectively, Figure 5a) with a moderate fat contribution in the acquired Z-spectrum (Figure 5c). Compared to the asymmetry analysis approach, the Lorentzian method (#3) and interpolation method (#4) produced the most similar pH maps, with comparable mean values and percentages of detected pixels, whereas the positive method (#1) and the linear method (#2) resulted in more acidic tumor pH values and less tumor pH coverage (Figure 5b,d,e).

**Figure 6:**
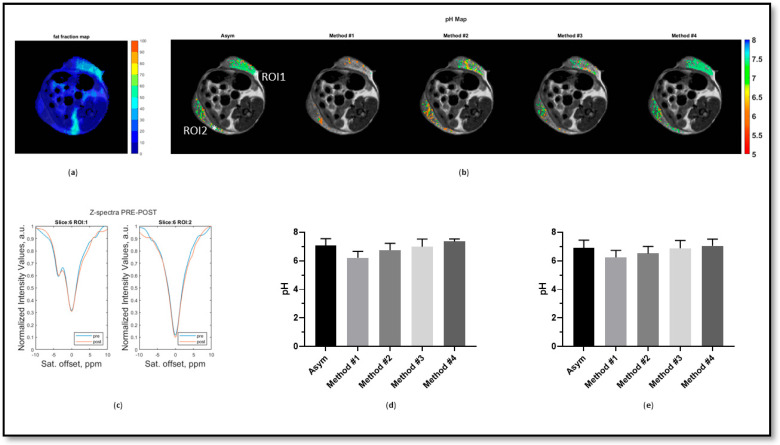
The fat fraction map (**a**), calculated tumor pH maps (**b**), Z-spectra before (blue) and after (red) iopamidol injection for the two ROIs (**c**), and tumor pH mean ± standard deviation values for ROI 1 (**d**) and ROI 2 (**e**) calculated for all the investigated methods in a tumor-bearing mouse with a high amount of fat.

The PyMT tumors showed moderate to high percentages of fat (ca. 29% and 10% for RO1 and RO2, respectively, Figure 6a), with a marked fat peak clearly visible in the acquired Z-spectrum for ROI1, in contrast to ROI2 (Figure 6c). Therefore, the difference in the fat contribution within the same tumor type allows a proper evaluation of the proposed fat correction methods. The asymmetry analysis provided acidic tumor pH values inside the tumor with a lower fat content (ROI 2), with only the Lorentzian method (#3) providing comparable pH values, whereas all the other approaches resulted in tumor pH values that were too acidic (positive and linear methods (#1 and #2), Figure 6e). For the tumor with high fat content (ROI1), the asymmetry analysis was strongly affected, showing less acidic tumor pH values (Figure 6b,d). Only the Lorentzian method (#3) provided accurate tumor pH values, similar to tumor pH values for ROI2. In contrast, all the other approaches resulted in tumor pH values that were too acidic (positive and linear methods (#1 and #2)) or closer to neutral values (interpolation method (#4), Figure 6b,d).

For the in vivo analysis, more than 4400 Z-spectra were processed with the proposed methods with a ASUSTeK D520MT (ASUS, Taipei, Taiwan) desktop pc running Windows 10 Pro equipped with an Intel Core i5-6400 CPU at 2.70 GHz and 16 GB of RAM. Among the investigated methods, the Lorentzian method (#3) was the slowest, requiring ca. 5650 s to complete the analysis. On the other hand, all the other methods were faster, with comparable acquisition times below 10 min (458 s, 470 s, 530 s and 460 s for the asymmetry analysis, positive method (#1), linear method (#2) and interpolation method (#4), respectively).

## 4. Discussion

The presence of lipids in varying amounts inside tissues can potentially affect the shape of the acquired Z-spectra and hence lead to erroneous CEST contrast quantification. Even the presence of low fat fractions can have an effect on the shape of the measured Z-spectrum, as shown in Figure 3a. To compensate for the lipid-induced inaccuracy in the MTRasym calculation, we investigated four different post-processing methods and evaluated them both in vitro and in vivo. All the methods aim to reduce or compensate the overall contribution of the lipid signal on the negative side of the acquired Z-spectrum, thus removing the main source of the erroneous CEST contrast.

In the phantom experiments, all the methods but #2 were able to successfully remove the lipid contribution even with large fat fraction values, with the Lorentzian method (#3) providing the best results in terms of pH accuracy and detectability. Also, the linear and interpolation methods (#2 and #4) demonstrated accurate pH calculation with low to moderate (10–30%) or moderate to high (30–60%) *FF* values (Figure 4c); however, they showed higher pH variations with increasing fat fraction values (Figure 4e), with the interpolation method (#4) being the most affected.

For the in vivo experiments, we investigated tumors with different amounts of fat to further validate the proposed methods. In the presence of low *FF* values, most of the methods are able to successfully remove the small lipid contribution, hence leading to comparable tumor pH maps similar to those provided by the conventional asymmetry analysis. The calculated average values and spatial distributions were similar across the asymmetry analysis approach and the Lorentzian and interpolation methods (#3 and #4) (Figure 5d,e). The linear method (#2) did not succeed in vivo in removing the lipid contribution, as observed for the in vitro data. However, in contrast to the in vitro findings, the positive method (#1) was unable to provide accurate pH measurements even with low *FF* values.

On the other hand, in the presence of tissues with high fat fraction values, the asymmetry analysis showed incorrect (less acidic) tumor pH values (ROI 1, Figure 6), and only the Lorentzian method (#3) could robustly remove the lipid contribution, therefore providing reliable tumor pH maps. All the other methods, including the interpolation method (#4), provided less accurate tumor pH values with increasing fat content. Overall, among the proposed methods, the Lorentzian method (#3) stands out as the most robust approach. One tentative explanation is based on the effect of the fat content on the overall shape of the Z-spectrum. With moderate fat content, both the conventional asymmetry analysis and the positive method (#2) are not robust enough to compensate for this disturbance. On the other hand, the linear method (#2) likely removes too much information from the Z-spectrum, which is better maintained by the Lorentzian method (#3), hence providing more accurate pH results.

Fat suppression methods throughout selective saturation are designed to reduce the fat signal but do not guarantee complete fat signal suppression, especially when the fat fraction exceeds 40%. This limitation could arise from the sub-optimal power of the fat suppression pulse or be related to B_0_ inhomogeneities that reduce the efficiency of the fat suppression scheme. In fact, in previous studies, lipid fat correction methods were able to completely remove lipid artifacts in tissues with low fat fractions, whereas the residual fat signal was not successfully removed in the presence of very high fat fraction values. In the study by Zhang and coworkers in healthy subjects, successful water–fat separation was achieved, although in some of the pixels with a high fat fraction (approximately >50%), the lipid peak was still detectable in the water-only images [96]. In another study by Zimmermann et al., an extensive examination of the Z-spectra at various fat fraction values indicated that the maximum evaluable *FF* for the in vivo acquisition protocol was approximately 50%. Voxels characterized by *FF* percentages exceeding 50% were omitted from the analysis [43]. Although the Lorentzian method (#3) is not influenced by the lipid signal up to a fat fraction of ca. 50–60%, thus providing accurate tumor pH measurements, higher fat fractions can still lead to uncorrected lipid artifacts and hence wrong CEST contrast calculations. However, in tumor tissues, although moderate fat fractions can be observed, they are usually low and hence successfully corrected by the proposed methods. On the other hand, very high fat fraction values are only observed in breasts, where fibroglandular and fat tissues are usually interleaved, resulting in a high fat signal. Even in the investigated transgenic PyMT breast cancer murine model, where cancer lesions develop within the mammary glands, the observed fat fraction was below the limit that the investigated Lorentzian method (#3) is able to successfully correct.

## 5. Conclusions

In conclusion, several approaches to accurately calculate CEST contrast even in the presence of fat were implemented and evaluated. In vitro and in vivo studies showed the capability of these approaches to calculate the CEST contrast and then to accurately measure tumor pH without requiring complex acquisition schemes that would further increase the overall acquisition time. Among the different methods, the approach to calculating the CEST contrast by replacing the negative part of the spectrum with the water pool contribution upon fitting the Z-spectrum with the Lorentzian method (#3) produced the best results in terms of pH measurement and pixel detection both in vitro and in vivo with moderate to high fat contents.

## Data Availability

All data generated or analyzed during this study are included in this published article. The raw data used and/or analyzed during the current study are available within the GitHub repository at the following link: https://github.com/cim-unito/Fat-Correction (accessed on 24 June 2024).

## Supplementary Materials

The following supporting information can be downloaded at https://www.mdpi.com/article/10.3390/jimaging10070166/s1: Figure S1: Graphical description of method #1; Figure S2: Graphical description of method #2; Figure S3: Graphical description of method #3; Figure S4: Ratiometric values calculated at several fat fraction percentages with different titrated pH values and implementation of method #4; Figure S5: Z-spectra of iopamidol 30 mM titrated at pH = 6.4 at several percentages of fat values in the range 0–80% [106,107].
