# Supplementary material for: Development and Validation of Four Different Methods to Improve MRI-CEST Tumor pH Mapping in Presence of Fat
Source: J Imaging. 2024 Jul 12;10(7):166. doi: 10.3390/jimaging10070166 (PMC11277679; doi:10.3390/jimaging10070166)
Supplement: Supplementary file 1 [file jimaging-10-00166-s001.zip › jimaging-3025158-supplementary.pdf]

Supplementary Materials

Development and Validation of Four Different Methods to Improve MRI-CEST Tumor pH Mapping in Presence of Fat

Francesco Gammaraccio [1,†], Daisy Villano [1,†], Pietro Irrera [2], Annasofia A. Anemone [1], Antonella Carella [2], Alessia Corrado [2] and Dario Livio Longo [2,*]

In this section, we provide a detailed description of the four developed methods:

Positive Method (#1)

The positive method (#1) calculates the contrast considering only the positive part of the Z-spectrum using equation #2 [1]. The analysis was performed assuming that the water signal intensity without any saturation is equal to 1 ($S_0 = 1$). Equation #2 can be rewritten as follows:

$$ST = 1 - S_{\Delta\omega}$$

where $S_{\Delta\omega}$ is the water signal intensity in the presence of a saturation pulse at an offset Δω (i.e., Δω = 4.2 ppm and 5.5 ppm for iopamidol). In Figure S1, a graphical representation of the CEST contrast calculation using only the positive part of the spectrum is shown.

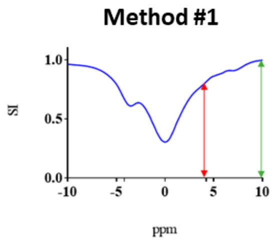

$$ST_{\Delta\omega} = \frac{S_0 - S_{\Delta\omega}}{S_0}$$

Figure S1. The positive method (#1). The blue curve represents a hypothetical Z-spectrum affected by the presence of fat. The red bar represents the water signal intensity in the presence of a saturation pulse at an offset Δω (i.e., Δω = 4.2 ppm and 5.5 ppm for iopamidol). The green bar is the water signal intensity without any saturation, which is assumed to be equal to 1.

Linear Method (#2)

The linear method (#2) involves replacing the contribution of the fat signal, which is assumed to be between -2 and -5.9 ppm for in vivo analysis and between -2 and -7.5 ppm for in vitro analysis, using a linear interpolation via the Matlab function 'interp1' (Figure S2). The CEST contrast is then calculated by asymmetry analysis using equation #1 [2].

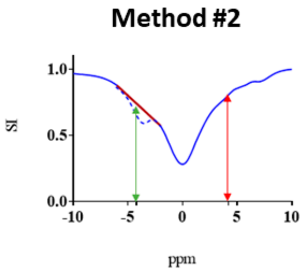

$$\mathrm{ST}_{\Delta\omega} = \frac{S_{-\Delta\omega} - S_{\Delta\omega}}{S_{-\Delta\omega}}$$

Figure S2. The linear method (#2). The fat contribution, represented by the dashed blue line, has been replaced with a linear interpolation, modeled by the solid red line.

Lorentzian Method (#3)

The Lorentzian method (#3) consists of replacing the negative part of the Z-spectrum with the water pool contribution upon the Lorentzian fitting of the spectrum. Lorentzian CEST curve fitting was implemented with the open-source Matlab-based code (https://github.com/cest-sources/CEST_EVAL, last accessed on 15/10/2023). The resulting Z-spectrum is characterized by two components: a negative contribution from Lorentzian CEST curve fitting and a positive contribution from the acquired Z-spectrum (Figure S3). Based on this resulting spectrum, the contrast is then calculated by asymmetry analysis using equation #1.

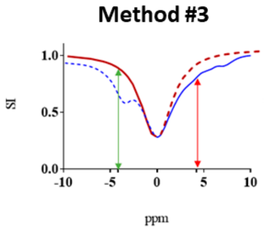

$$\mathrm{ST}_{\Delta\omega} = \frac{S_{-\Delta\omega} - S_{\Delta\omega}}{S_{-\Delta\omega}}$$

Figure S3. The Lorentzian method (#3). The blue curve represents the acquired Z-spectrum, while the red curve represents Lorentzian CEST curve fitting. The resulting spectrum consists of a negative component, represented by the solid red curve, and a positive component, represented by the solid blue curve.

Interpolation Method (#4)

The calculated CEST parametric maps for the ST contrast at 4.2 and 5.5 ppm of the phantom with pH of 6.4 and 6.9 are obtained by evaluating voxel-wise saturation transfer at specific offsets using asymmetric analysis (equation #1). The ratiometric map is calculated by performing a voxel-wise division of the contrast values obtained at 4.2 ppm and 5.5 ppm, as described in equation #4. Figure S4a displays the resulting ratiometric values and fat fraction values obtained from the respective maps. Subsequently, cubic smoothing spline interpolation was applied to the data using the Matlab function 'csaps', where the smoothing parameter p is equal to 0.01 (p = 0.01).

A series of interpolated curves (n=200) were generated between the two original curves using the Matlab function 'linspace' (Figure S4b). This interpolation was performed to calculate the ratiometric value based on the experimental fat fraction value and then to

correct it, assuming a fat fraction of zero (Figure S4c). The corrected ratiometric value is used to calculate the pH value via a calibration curve (Figure S4d) [103].

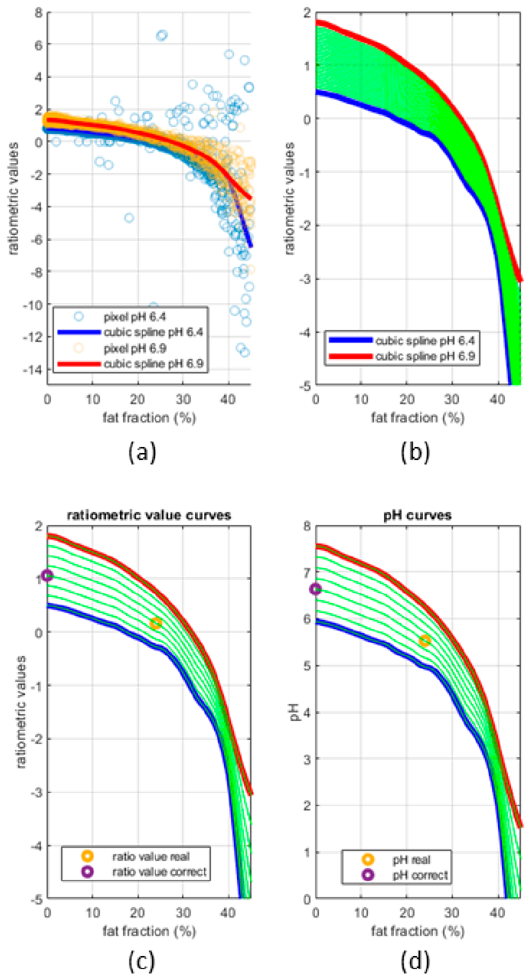

Figure S4. (a) Fat fraction pixel values on the x-axis and ratiometric values on the y-axis for the phantom with pH of 6.4 and 6.9. Blue represents the phantom with a pH of 6.4, while red indicates the phantom with a pH of 6.9. Pixels with fat fraction values up to 45% are included. The fat fraction values were adjusted by subtracting the average calculated within ROI 1, which contains pixels with the lowest fat fraction. Specifically, for the phantom at pH 6.4, the average fat fraction is 2.02, and for the phantom at pH 6.9, it is 3.38. (b) The two interpolation curves were shifted vertically so that the pH 6.4 phantom's curve has a ratiometric value of 0.5 when FF=0, and the pH 6.9 phantom's curve has a ratiometric value of 1.8 when FF=0. A set of interpolated curves was generated between these two curves (n=200). (c) A set of interpolated curves with n=8 for enhanced visual representation. The yellow circle marks the real ratiometric value for a 25% fat fraction, while the purple circle indicates the ratiometric value after applying the correction factor. (d) pH curves derived from the ratiometric value curves using a calibration curve. The yellow circle marks the real pH value for a 25% fat fraction, while the purple circle indicates the pH value after applying the correction factor.

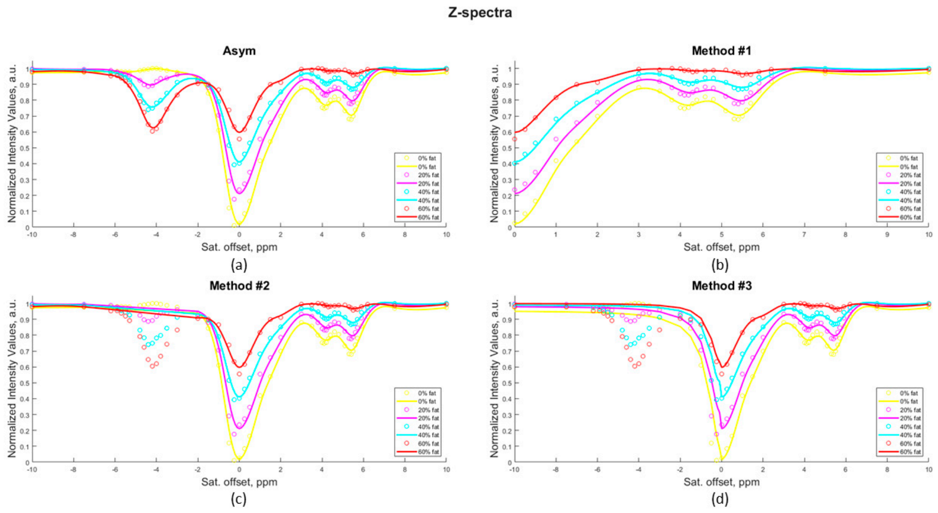

Figure S5. In vitro Z-spectra of iopamidol 30 mM titrated at pH = 6.4 at several percentages of fat in the range 0-60% and graphical representations of the proposed methods for fat correction. Asymmetric analysis utilizes the original spectrum (a), method #1 considers only the positive part of the spectrum (b), method #2 replaces the negative part of the Z-spectrum with a linear interpolation (c) and method #3 consists of replacing the negative part of the Z-spectrum with the water pool contribution upon Lorentzian fitting of the spectrum (d).

References

1. Terreno, E.; Stancanello, J.; Longo, D.; Castelli, D.D.; Milone, L.; Sanders, H. M. H. F.; Kok, M. B.; Uggeri, F.; Aime, S. Methods for an improved detection of the MRI-CEST effect. CMMI. 2009, 4, 237–247. https://pubmed.ncbi.nlm.nih.gov/19839029/.
2. Kim, J.; Wu, Y.; Guo, Y.; Zheng, H.; Sun, P. Z. A review of optimization and quantification techniques for chemical exchange saturation transfer MRI toward sensitive in vivo imaging. CMMI. 10, 163–178. doi:10.1002/cmmi.1628 (2015).
